# The effect of field strength on glioblastoma multiforme response in patients treated with the NovoTTF™-100A system

**DOI:** 10.1186/1477-7819-12-162

**Published:** 2014-05-22

**Authors:** Scott G Turner, Thomas Gergel, Hueizhi Wu, Michel Lacroix, Steven A Toms

**Affiliations:** 1 Geisinger Medical Center, 17822 Danville, PA, USA; 2 Geisinger Wyoming Valley, 18702 Wilkes-Barre, PA, USA

**Keywords:** glioblastoma, tumor treating fields, cytotoxic, NovoTTF therapy, NovoTTF-100A System

## Abstract

The NovoTTF™-100A system is a portable device that delivers intermediate frequency alternating electric fields (TTFields, tumor treating fields) through transducer arrays arranged on the scalp. An ongoing trial is assessing its efficacy for newly diagnosed glioblastoma multiforme (GBM) and it has been FDA-approved for recurrent GBM.

The fields are believed to interfere with formation of the mitotic spindle as well as to affect polar molecules at telophase, thus preventing cell division. The position of the four arrays is unique to each patient and optimized based on the patient’s imaging. We present three patients with GBM in whom the fields were adjusted at recurrence and the effects of each adjustment. We believe there may be a higher risk of treatment failure on the edges of the field where the field strength may be lower.

The first patient underwent subtotal resection, radiotherapy with temozolomide (TMZ), and then began NovoTTF Therapy with metronomic TMZ. She had good control for nine months; however, new bifrontal lesions developed, and her fields were adjusted with a subsequent radiographic response. Over the next five months, her tumor burden increased and death was preceded by a right insular recurrence.

A second patient underwent two resections followed by radiotherapy/TMZ and NovoTTF Therapy/TMZ. Six months later, two new distal lesions were noted, and he underwent further resection with adjustment of his fields. He remained stable over the subsequent year on NovoTTF Therapy and bevacizumab.

A third patient on NovoTTF Therapy/TMZ remained stable for two years but developed a small, slow growing enhancing lesion, which was resected, and his fields were adjusted accordingly. Interestingly, the pathology showed giant cell GBM with multiple syncitial-type cells.

Based on these observations, we believe that field strength may play a role in ‘out of field’ recurrences and that either the presence of a certain field strength may select for cells that are of a different size or that tumor cells may change size to avoid the effects of the TTFields.

## Background

Glioblastoma multiforme (GBM) remains a fatal disease that affects more than 10,000 people in the United States each year [1]. Standard therapy currently consists of surgical resection, when possible, followed by radio- and chemotherapy. In 2005, Stupp *et al*. [2] demonstrated a significant improvement in progression-free survival at 6 months (PFS-6) and overall survival (OS) when temozolomide, an alkylating agent, was given during radiotherapy and for six months following radiation. More recently, bevacizumab, a monoclonal antibody against VEGF, has proven effective in treating patients with recurrent GBM [3]. Much interest exists regarding treatment options following progression on bevacizumab, one of which is the NovoTTF-100A system, which was FDA-approved in 2011 for adult patients with recurrent GBM following surgery and chemotherapy [4]. This modality utilizes a novel mechanism to target tumor cells with minimal local side effects.

Living cells contain many polar molecules that exert and respond to electric fields. Low frequency (<1 kHz) fields induce membrane depolarization, as seen in nerve and muscle cells, whereas fields in the MHz range generate heat and are used in radiofrequency ablation and diathermy [5]. Low- to moderate-frequency fields (kHz to MHz) were thought to be biologically inert, but have been shown to cause cell rotation [6] and alignment of microscopic particles [7]. Kirson *et al*. determined that intermediate-frequency alternating fields exert these effects on tumor cells *in vitro*[8]. Cells become polarized during mitosis, and dividing cells tend to orient along the direction of an externally applied field. In addition, field intensity is increased at the cleavage furrow, which draws polar intracellular particles toward the furrow, which interferes with cytokinesis. At an intracellular level, assembly and disassembly of microtubules are disrupted in the presence of these fields due to changes in alignment of tubulin dimers that may prevent polymerization.

**Figure 1:**
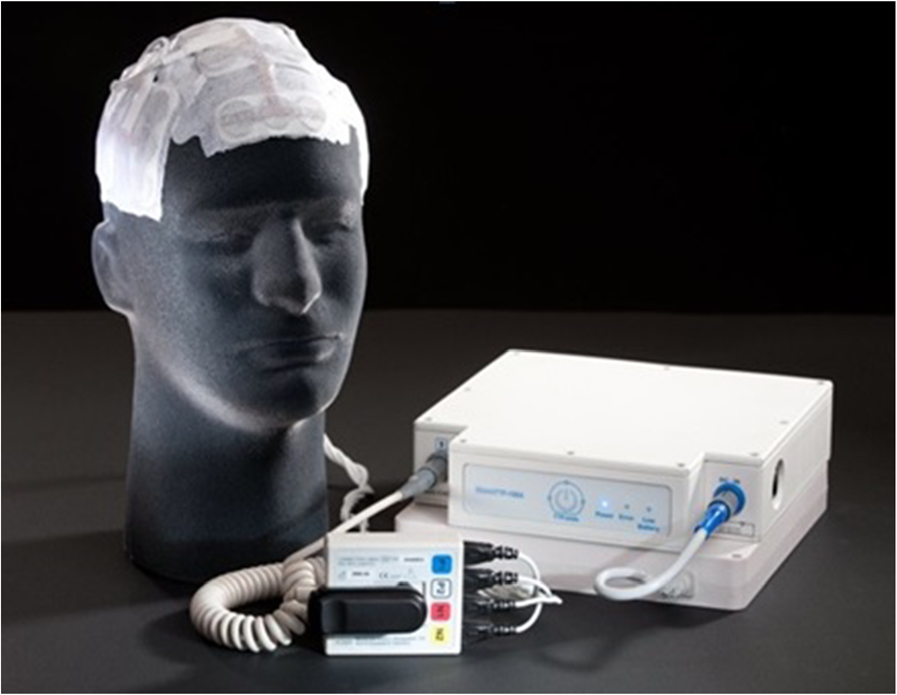
The NovoTTF-100A system.

The device used with Novo TTF Therapy delivers electric fields alternating in direction through transducer arrays arranged on the scalp (Figure 1). Field strength, direction, and electrode position are important to optimize during treatment. Kirson *et al*. examined the effects of TTF on several human and animal tumor types *in vitro* and found that cell proliferation slowed during exposure to the fields and that the effect was dependent on the field frequency. The 200-kHz fields were the most cytotoxic to rat glioma (F-98) cells. When glioma cells were implanted intracranially into Fischer rats and treated with 200 kHz fields, it was found that fields applied in multiple directions are optimal for cytotoxicity since cells divide along random planes [9]. In our patients, as per the instructions for use, we use four arrays that are positioned on the scalp based on the patient’s imaging; these arrays deliver alternating fields along two planes in order to maximize delivery of the electric field to the tumor. By taking into account the different impedances of bone, skin, brain and CSF, Kirson et el. used computer modeling to demonstrate the distribution of field intensity within the brain [9]. Each patient’s magnetic resonance imaging (MRI) is used to individualize therapy to maximize delivery of the TTField to the region of the tumor, while other regions of the brain receive fewer volts per centimeter. More recently, the NovoTAL system has been developed for investigators to calculate the optimal placement of the electrodes on the scalp based on each of their patient’s imaging. We present three patients in whom the field location was adjusted after recurrence to better treat the new lesion based on updated imaging and the results of these adjustments.

## Case presentation

### Case 1

**Figure 2:**
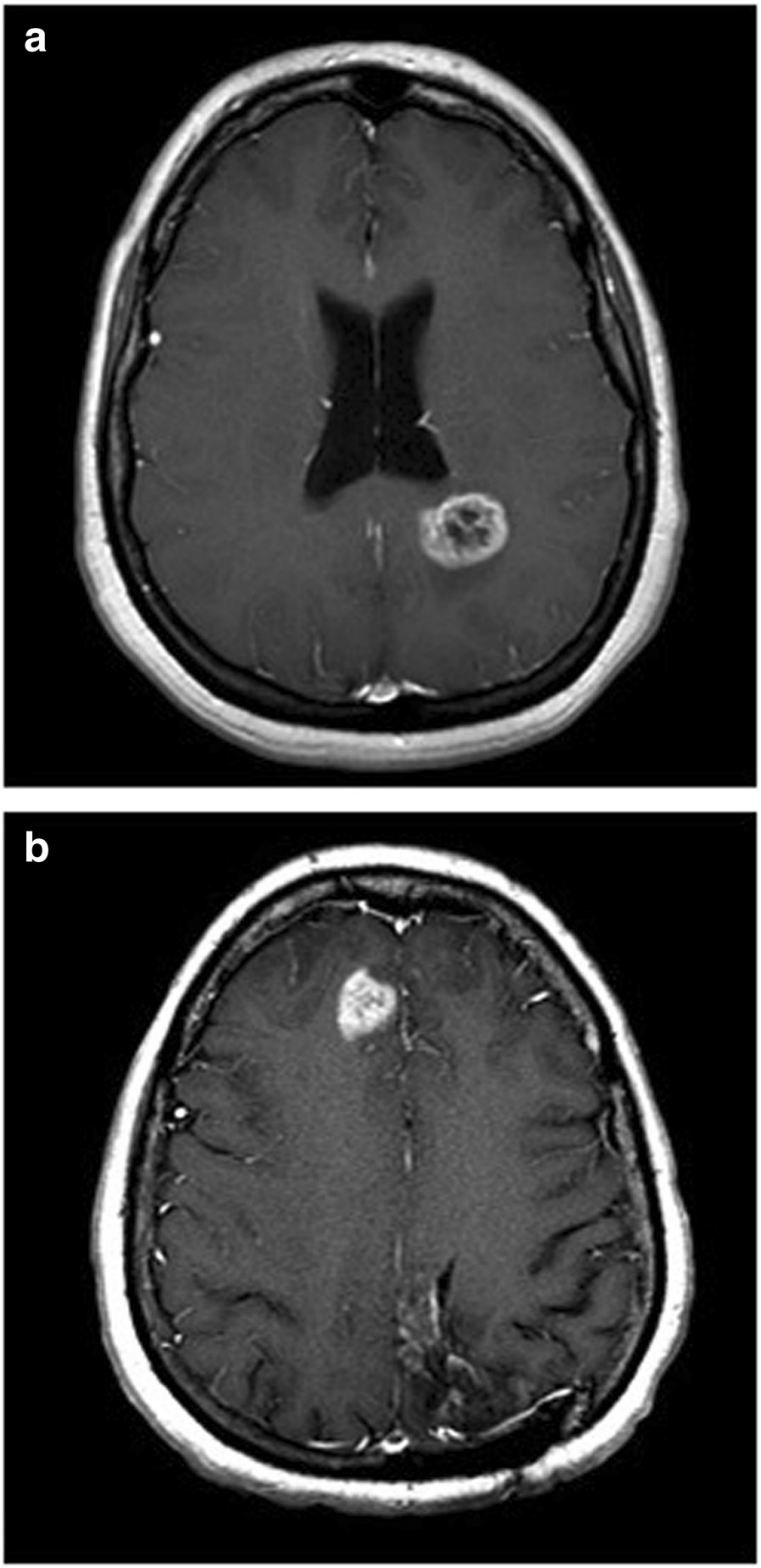
**Patient 1 imaging.** MRI showing **(a)** initial left parietal glioblastoma multiforme (GBM) and **(b)** bifrontal recurrence.

A 39-year-old woman who presented after a new-onset seizure was found to have a left parietal GBM (genetics not available) (Figure 2a). She underwent subtotal resection followed by radiotherapy with daily temozolomide (TMZ), and then NovoTTF Therapy with metronomic TMZ. After 3 months, her MRI showed progression near her initial resection site, and temozolomide was replaced by bevacizumab (BEV), with NovoTTF Therapy continuing. Nine months later, she had local recurrence in addition to bifrontal enhancing lesions (Figure 2b), and her TTFields were adjusted. Over the next five months, her multifocal tumor burden increased, and death was preceded by a right insular recurrence.

### Case 2

**Figure 3:**
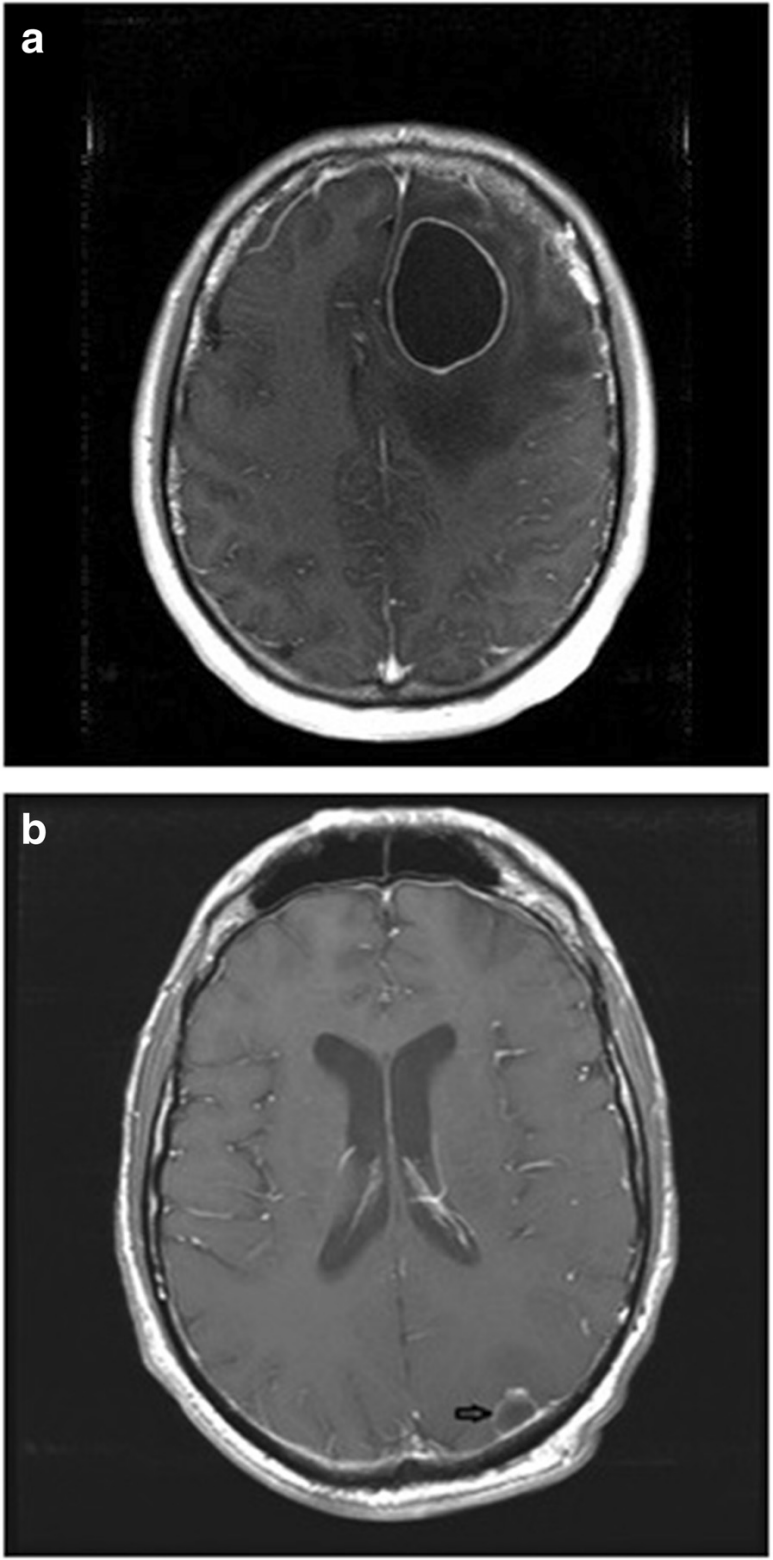
**Patient 2 imaging.** MRI showing **(a)** left frontal glioblastoma multiforme (GBM) and **(b)** subsequent left parietal recurrence (arrow).

A 41-year-old man presented after a week of intermittent headaches and was found to have a left frontal enhancing mass (Figure 3a). A gross total resection was performed and the pathology showed GBM with primitive neuroectodermal tumor (PNET) component (MGMT promoter unmethylated, EGFR not amplified, 1p/19q co-deletion negative). Surgery was followed by radiotherapy and daily TMZ and then NovoTTF therapy/metronomic TMZ. Six months later, a distal lesion were noted (Figure 3b), and he underwent further resection with adjustment of his fields. In order to address the PNET component if his tumor, and because a combination of ifosfamide, carboplatin, etoposide (ICE) has been shown to be effective against this tumor type [10], his chemotherapy was changed to bevacizumab with ICE (ifosfamide, carboplatin, etoposide) × six cycles. He continued on bevacizumab with NovoTTF therapy for over a year until he developed leptomeningeal disease and passed away in July 2013.

### Case 3

**Figure 4:**
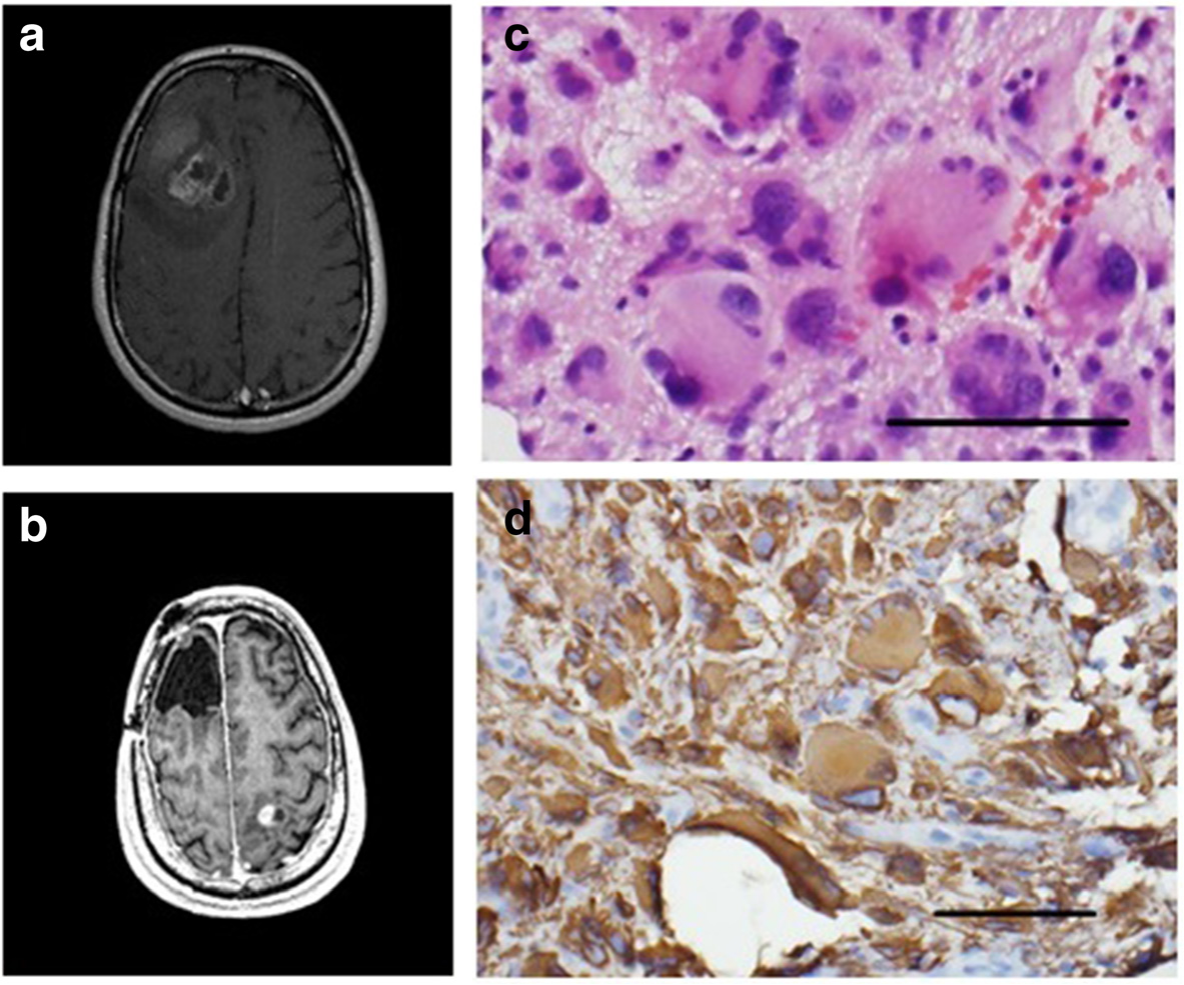
**Patient 3 imaging.** MRI showing **(a)** initial right frontal glioblastoma multiforme (GBM) and **(b)** recurrent left parietal GBM which had a giant cell component. **(c)** Hematoxylin and eosin (H&E) stain showing giant cells and **(d)** glial fibrillary acidic protein (GFAP) immunostain (scale bar = 0.1 mm).

A 45-year-old man presented with headaches and was found to have a right frontal GBM (MGMT promoter unmethylated, EGFR not amplified, 1p/19q codeletion status unknown) (Figure 4a), for which he had a gross total resection followed by radiation and daily TMZ and then metronomic TMZ with NovoTTF therapy. He remained neurologically intact with no radiographic evidence of recurrence for 22 months. A surveillance MRI revealed a small enhancing lesion in the left parietal lobe (Figure 4b), which was resected, and his fields were adjusted accordingly. The histology of the recurrent lesion showed multiple giant syncytial-type cells, and he has remained in good health over the subsequent nine months.

## Conclusions

Given its poor prognosis, new therapies to treat GBM are desperately needed. The NovoTTF-100A System offers a treatment modality with minimal local side effects and no known systemic side effects, whose efficacy is comparable to other chemotherapy regimens [4]. In fact, combining chemotherapy with NovoTTF therapy may improve efficacy without added toxicity [11]. In order to optimize this response, the field strength and orientation must be taken into account; the positioning of the electrodes is based upon the patient’s MRI in order to optimize delivery of the field to the tumor.

Our three cases demonstrate that, when a tumor progresses locally, adjustment of the fields may lead to a better response; however, multifocal disease is difficult to optimally treat despite this adjustment, as seen in our first patient. NovoTTF therapy failure leading to remote recurrence in the second two patients may have been due to lower field strength at these distal sites based upon the position of the electrodes; as the field was optimized in one location, the strength may have been lowered elsewhere below a threshold where newly seeded tumor cells were able to divide.

Following resection of the recurrent lesion in our third patient, we discovered that the cells had a ‘giant-cell’ morphology, leading us to hypothesize that giant cells avoided the toxicity of the TTFields or that existing cells adopted this giant cell phenotype, which may require different parameters to achieve cytotoxicity. Indeed, Kirson *et al*. [8] demonstrated that the optimal frequency for cytotoxicity *in vitro* differed in various tumor cell types and that optimal TTField frequency is inversely related to cell size [9]. Alternately, because this patient’s recurrence was distant from the original tumor site, the potentially decreased field strength may have also contributed to the recurrence, as we hypothesized for our other patients.

In addition to differences in TTField strength within the brain, other factors may be associated with distant recurrence including radiation field strength and genetic profile. Brandes *et al*. [12] found that, in 79 patients with recurrent GBM treated with radiation plus daily TMZ followed by metronomic TMZ, distant recurrence (outside the radiation field) occurred in 21.5% of patients, while recurrence within or at the radiotherapy margin occurred in 85% of patients with MGMT unmethylated promoters and 57.9% of patients with MGMT methylated promoters. Why distant recurrence should be associated with MGMT promoter methylation is not known but may be due to changes in cell motility patterns or the fact that the combination of radiation and temozolomide act synergistically on cells with MGMT promoter methylation, thus reducing the likelihood of local recurrence. All three patients presented here had recurrence outside their initial radiation treatment fields, and though genetics are not available for our first patient, the second two patients had unmethylated MGMT promoters and would therefore be thought less likely to recur distally, according to the findings described by Brandes *et al*.

Treatment with anti-angiogenic agents should also be taken into account when considering distal GBM recurrence. Bevacizumab has been shown to induce a more invasive phenotype *in vitro* and *in vivo*, perhaps by upregulating expression of matrix metalloproteinases [13]. In addition, clinical studies [14,15] have found that treatment with bevacizumab is associated with an increased incidence of distal recurrence. This may be due to genetic alterations that effect a more invasive phenotype, allowing cells to migrate along normal blood vessels or within the extracellular matrix in order to escape a hypoxic microenvironment [16]. The recurrences described in the first two patients presented here should therefore be considered in light of their treatment with bevacizumab. Further study is clearly warranted in order to better understand the subcellular effects produced by the TTFields, allowing us to tailor them to specific tumor cell sizes and types.

## Consent

Written informed consent was obtained from the patients for publication of this Case report and any accompanying images. A copy of the written consent is available for review by the Editor-in-Chief of this journal.

## Abbreviations

BEV: bevacizumab; EGFR: epidermal growth factor; GBM: glioblastoma multiforme; kHz: kilohertz; MGMT: methylguanine methyltransferase; MHz: megahertz; MRI: magnetic resonance imaging; OS: overall survival; PFS-6: progression-free survival at 6 months; PNET: primitive neuroectodermal tumor; TMZ: temozolomide; TTFields: tumor-treating fields.

## Competing interests

Dr. Steven A. Toms has consulting agreements with Medtronics and Novocure.

## Authors’ contributions

SGT, TG, ML, and SAT provided direct clinical patient care. HW provided pathologic analysis. SGT drafted the manuscript. All authors read and approved the final manuscript.
